# Supplementary material for: Association between oral microbiome and seven types of cancers in East Asian population: a two-sample Mendelian randomization analysis
Source: Front Mol Biosci. 2023 Nov 21;10:1327893. doi: 10.3389/fmolb.2023.1327893 (PMC10702768; doi:10.3389/fmolb.2023.1327893)
Supplement: Supplementary file 1 [file DataSheet1.ZIP › Supplementary Materials/MR plots for tongue/tongue═╝/Colorectal cancer/pheno.2468_to_colorectal cancer_forest.pdf]

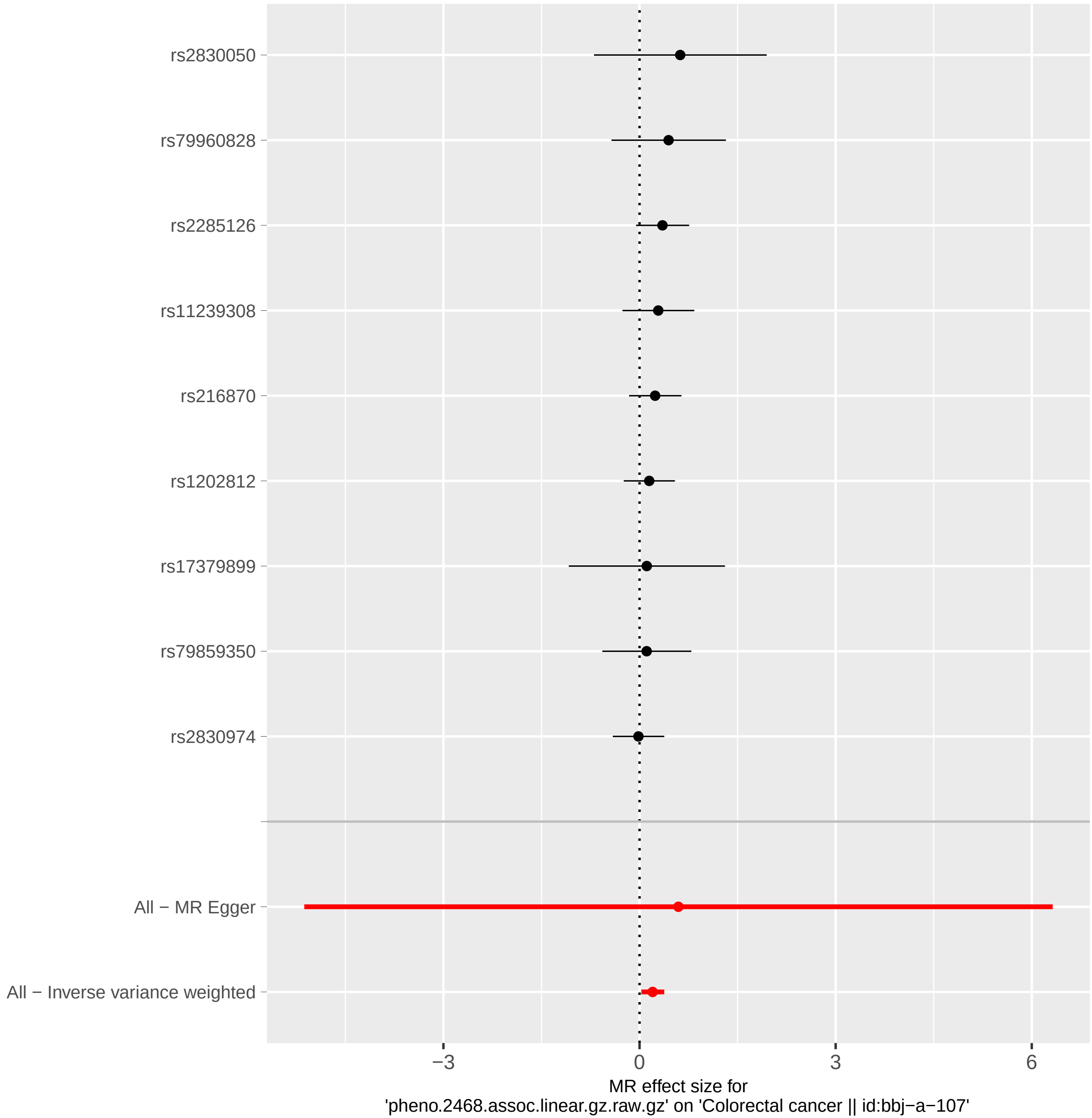

rs2830050
rs79960828
rs2285126
rs11239308
rs216870
rs1202812
rs17379899
rs79859350
rs2830974
All − MR Egger
All − Inverse variance weighted
−3
0
3
6
MR effect size for
'pheno.2468.assoc.linear.gz.raw.gz' on 'Colorectal cancer || id:bbj−a−107'
